# Methionine Restriction Prevents Lipopolysaccharide-Induced Acute Lung Injury via Modulating CSE/H_2_S Pathway

**DOI:** 10.3390/nu14020322

**Published:** 2022-01-13

**Authors:** Jiaxiang Duan, Lunli Xiang, Zhen Yang, Li Chen, Jianteng Gu, Kaizhi Lu, Daqing Ma, Hailin Zhao, Bin Yi, Hongwen Zhao, Jiaolin Ning

**Affiliations:** 1 Department of Anesthesia, Southwest Hospital, Third Military Medical University, Chongqing 400038, China; duanjiaxiang@tmmu.edu.cn (J.D.); yangzhen@tmmu.edu.cn (Z.Y.); jiantenggu@tmmu.edu.cn (J.G.); lukaizhi@tmmu.edu.cn (K.L.); 2 Department of Nephrology, Southwest Hospital, Third Military Medical University, Chongqing 400038, China; xianglunli@tmmu.edu.cn; 3 Department of Breast and Thyroid Surgery, Southwest Hospital, Third Military Medical University, Chongqing 400038, China; chenlili@tmmu.edu.cn; 4 Division of Anaesthetics, Pain Medicine and Intensive Care, Department of Surgery and Cancer, Faculty of Medicine, Imperial College London, Chelsea and Westminster Hospital, London SW10 9NH, UK; d.ma@imperial.ac.uk (D.M.); hailin.zhao06@imperial.ac.uk (H.Z.)

**Keywords:** methionine restriction, cystathionine-gamma-lyase, hydrogen sulfide, LPS, acute lung injury

## Abstract

Acute lung injury (ALI) and acute respiratory distress syndrome (ARDS) result in high mortality, whereas effective treatments are limited. Methionine restriction (MR) has been reported to offer various benefits against multiple pathological processes of organ injuries. However, it remains unknown whether MR has any potential therapeutic value for ALI/ARDS. The current study was set to investigate the therapeutic potential of MR on lipopolysaccharide (LPS)-induced ALI and its underlying mechanisms. We found that MR attenuated LPS-induced pulmonary edema, hemorrhage, atelectasis, and alveolar epithelial cell injuries in mice. MR upregulated cystathionine-gamma-lyase (CSE) expression and enhanced the production of hydrogen sulfide (H_2_S). MR also inhibited the activation of Toll-like receptors 4 (TLR4)/NF-κB/NOD-like receptor protein 3 (NLRP3), then reduced IL-1β, IL-6, and TNF-α release and immune cell infiltration. Moreover, the protective effects of MR on LPS-induced ALI were abrogated by inhibiting CSE, whereas exogenous H_2_S treatment alone mimicked the protective effects of MR in *Cse*^−/−^ mice after LPS administration. In conclusion, our findings showed that MR attenuated LPS-induced lung injury through CSE and H_2_S modulation. This work suggests that developing MR towards clinical use for ALI/ARDS patients may be a valuable strategy.

## 1. Introduction

Acute lung injury (ALI) and its severe form, acute respiratory distress syndrome (ARDS), is a devastating condition characterized by the accumulation of inflammatory cells, injury of the epithelial/capillary interface, increase in vascular permeability and pulmonary edema, and ultimate refractory hypoxia [1,2,3,4]. Gram-negative bacterium associated pathogenesis is one of the most common causes of ALI/ARDS [5]. Patients with this syndrome are often treated with respiratory support, antibiotics, and even extracorporeal membrane oxygenation. However, the mortality is still up to 40%, which is primarily attributed to the absence of effective treatments [6]. It is reported that methionine restriction (MR) can confer multiple lifespan and therapeutic benefits from yeast to mammals [7]. In yeast, flies, worms and fish, the primary benefit of MR is prolonging the lifespan. In mammals, MR enhances the capacity of stress resistance, improves metabolic fitness, postpones the loss of organ function, and prevents metabolism and aging associated with diseases such as diabetes, cardiovascular disease, and cancer [8,9,10,11,12]. MR has been shown to induce beneficial effects through multiple mechanisms [13]. It induces autophagy by inhibiting the mechanistic target of rapamycin complex 1 (Mtorc1) and increasing polyamine, decreases reactive oxygen species (ROS) production in mitochondria, and increases the production of hydrogen sulfide (H_2_S), a metabolic byproduct of sulfur-containing amino acid such as methionine, which acts as a gasotransmitter and plays an important protective role in various physiological and pathological processes including inflammation, apoptosis, and functional regulation of blood vessels [14,15,16,17,18].

Recently, a growing number of studies have reported that MR also improves acute disease conditions. For example, it attenuates renal ischemia/reperfusion-induced myocardial injury by activating the cystathionine-gamma-lyase (CSE)/H_2_S/endoplasmic reticulum stress (ERS) pathway in diabetic mice, and alleviates kidney injury by maintaining osmotic balance via upregulating genes (such as *Aqp2, Scnn1a* and *Slc6a19*) that are involved in ion transporters. In addition, it also improves dextran sodium sulfate (DSS)-induced colitis by upregulating catalase (CAT), superoxide dismutase (SOD), and glutathione peroxidase (GPx) activities; decreasing myeloperoxidase (MPO), tumor necrosis factor-α (TNF-α), and interleukin-1β (IL-1β); and reversing the activation of the nuclear factor kappa-B (NF-κB) signaling pathway [19,20,21].

Unlike the majority of vital organs, the lung is directly open to the air and constantly exposed to pathogens, allergens, irritants, and toxins; thus, in general, the lung has a high risk of being injured, whilst effective therapies for preventing and treating such injuries are not yet available. Therefore, the current study was set to investigate the effects of MR on lipopolysaccharide (LPS)-induced ALI in mice and explore the underlying mechanisms.

## 2. Materials and Methods

### 2.1. Animals

All experimental procedures were performed according to the Guide for the Care and Use of Laboratory Animals and approved by the ethical committee of the Third Military Medical University (IACUC approval number: AMUWEC20201908).

Male wild-type and cystathionine-gamma-lyase gene (*Cse*) knockout (*Cse^−/−^*) C57BL/6J mice (6–8 week, 20–25 g) were purchased from the Shanghai Model Organisms Center, Inc. (Shanghai, China). All mice were housed in the Third Military Medical University animal center under standard conditions at 23 ± 1 °C and 55 ± 10% humidity with a 12 h/12 h light/dark cycle. The animals had free access to a routine diet for 1 week before dietary intervention. Then the MR diet (0.43% Methionine) and control diet (0.86% Methionine) were given to the mice, respectively from 7 days before LPS administration to 3 days after LPS administration when they were sacrificed. The dietary composition is provided in Supplementary Table S1. The body weight was monitored every day. The mice were euthanized and their blood samples were collected, and their lung tissue samples and plasma were stored at −80 °C for further experiments.

### 2.2. LPS-Induced ALI Model and Drugs Administration

Mice were anesthetized by inhalation of 4% isoflurane (Abbott, Chicago, IL, USA). ALI was induced with an intratracheal instillation of 15 mg/kg (for an evaluation of lung injury) Lipopolysaccharide (LPS) (L2880, Sigma-Aldrich, St. Louis, MO, USA) or 25 mg/kg LPS (for an analysis of survival rate) in saline. The mice in the control group were instilled intratracheally with the same volume of saline. Some cohorts were injected intraperitoneally with Morpholin-4-ium 4-methoxyphenyl (morpholino) phosphinodithioate (GYY4137) (SB06, Dojindo, Kumamoto, Japan) (50 mg/kg) [22] in saline daily from 5 days before LPS administration to the third day after LPS administration; or injected with DL-Propargylglycine (PAG; 25 mg/kg) (P7888, Sigma-Aldrich, St. Louis, MO, USA) [23] in saline daily from 2 h before LPS administration to the third day after LPS administration.

### 2.3. IL-1β, IL-6 and TNF-α Measurement in Bronchoalveolar Lavage Fluids

Bronchoalveolar lavage fluid (BALF) was collected as described previously [24]. Briefly, 1 mL of pre-cooled sterile PBS buffer was injected slowly into bronchia and lungs via a tracheal cannula and extracted slowly after 5 min. The process was repeated three times. The BALF was collected and centrifuged at 4 °C, 3000 rpm for 20 min, and the supernatant was collected for further experiments. Then the levels of IL-1β (catalog no. H002), IL-6 (catalog no. H007-1-2) and TNF-α (catalog no. H052-1) were measured by an enzyme-linked immunosorbent assay (ELISA) according to the instructions of the manufacturer (Nanjing Jiancheng Bioengineering Institute, Nanjing, China).

### 2.4. Histopathology

The lungs were fixed in 4% paraformaldehyde for 24 h, dehydrated, embedded in paraffin, cut into 4 μm sections, and stained with hematoxylin and eosin (catalog no. G1120, Solarbio, Beijing, China). Pathological changes of the lungs were recorded by using a light microscope (Carl Zeiss, Shanghai, China). Micrographs of the lung sections were taken by a light microscope (Carl Zeiss, Shanghai, China) and assessed in a blinded manner with pathological changes of cells infiltration, edema, hemorrhage, and atelectasis, which were quantified according to our previously established protocol, ranging from 0 (minimal or no damage) to 3 (severe damage) [25].

### 2.5. Immunochemistry

Lung paraffin sections were deparaffinized, hydrated, repaired antigen, blocked with 10% goat serum (catalog no. SL038, Solarbio, Beijing, China), and incubated with primary antibodies (listed in Supplementary Table S2) at 4 °C overnight, as well as horseradish peroxidase-conjugated secondary antibodies (ZB-2301, goat anti-rabbit IgG H&L, 1:500, Zsbio, Beijing, China) for 1 h. The Diaminobenzidine Peroxidase Substrate Kit (catalog no. ZLI-9017, Zsbio, Beijing, China) was used to detect positive signals, and then counterstained with hematoxylin (catalog no. G1080, Solarbio, Beijing, China). Section microphases were taken by a light microscope and quantitatively analyzed with the Image-Pro plus 6.0 software. In order to analyze the intensity of proteins that were expressed in cells or on cell membranes including Aquaporin-5 (AQP5), Surfactant protein C (SFTPC), Receptor-interacting protein kinase 3 (RIPK3), NOD-like receptor protein 3 (NLRP3), CSE, cystathionine-beta-synthase (CBS), 3-mercaptopyruvate sulfurtransferase (MST), and toll-like receptors 4 (TLR4), the relative intensity was calculated by the total intensity in one arbitrary alveoli divided by the total area of cells in the region relative to the control group. For NF-κB, the relative intensity was calculated by the total intensity in nuclei of one arbitrary alveoli divided by the total area of nuclei in the region relative to the control group. The number of macrophages and neutrophils were counted by adhesion G protein-coupled receptor E1 (F4/80)- and lymphocyte antigen 6 complex (LY6G)-positive cells in one arbitrary alveoli [26,27].

### 2.6. H_2_S Measurement in Plasma and Lung Tissues

The H_2_S concentration in the plasma and lung were measured as described previously [28,29]. Briefly, the plasma were collected and centrifuged at 4 °C, 3000 rpm for 20 min. Lung tissue was homogenized (Ultra-Turrax) in 100 mM ice-cold potassium phosphate buffer (PH 7.4) and centrifuged at 4 °C, 12,000 rpm for 10 min. Plasma or lung homogenate supernatant both in 500 μL were mixed with 250 μL of distilled water and 250 μL of zinc acetate (1% *W*/*V*) in a test tube. Then, 133 μL of *N*,*N*-dimethyl-p-phenylenediamine sulfate (20 μM) in 7.2 mM HCl and 133 μL of FeCl_3_ (30 μM) in 1.2 mM HCl were also added to the test tube, and then incubated for 20 min at room temperature for coloration. Then, 250 μL of trichloroacetic acid (10% *W*/*V*) was added. The mixture was centrifuged at 4 °C, 6000 rpm for 5 min. The supernatant was collected and read by a spectrophotometer (Thermo Fisher Scientific, Shanghai, China) at 670 nm. The concentration of H_2_S was calculated against a calibration curve of Sodium Hydrosulphide (NaHS, 3.12–250 µM, Sigma, USA). The H_2_S concentration is expressed as nmol/mg of lung or nmol/mL of plasma.

### 2.7. Real-Time Quantitative PCR

Total RNA was extracted from lung tissues with the Trizol kit (catalog no. 12183555, Invitrogen, Carlsbad, CA) according to the manufacturer’s instructions. RNA purity and quantity were determined by a NanoDrop spectrophotometer (Thermo Fisher Scientific, Shanghai, China). Reverse transcription reactions were performed with the PrimeScript™ RT reagent kit (catalog no. RR037A, Takara, Otsu, Japan) and RT-qPCR was carried out with the SYBR Green PCR kit (catalog no. 204143, Qiagen, Hilden, Germany) on a CFX96™ Real-Time System (Bio-Rad, Shanghai, China). The expression of target genes was normalized to the GAPDH gene and the relative expression of target genes was calculated by the 2^−ΔΔCT^ method. The primer sequences were the following: *Cse*, forward, 5′- GTGGGACAAGAGCCTGAGCAAT-3′; reverse, 5′-GGATTTCCAGAGCGGCTGTATTC-3′. *Gapdh*, forward, 5′-AGGTCGGTGTGAACGGATTTG-3′; reverse, 5′-TGTAGACCATGTAGTTGAGGTCA-3′.

### 2.8. Statistical Analysis

Data were analyzed with the GraphPad Prism software (eighth edition). Data normality was tested by D’Agostino and Pearson and Shapiro–Wilk tests. If normally distributed, data were expressed as means ± SEM; otherwise, as the median (IQR). The normally distributed data were analyzed by an unpaired t-test (two groups) or one-way ANOVA (≥3 groups) followed by post hoc Tukey’s or Dunnett’s tests for multiple comparisons, while non-normally distributed data were analyzed by a nonparametric test (Kruskal-Wallis test and Dunn’s multiple comparisons test). Survival analysis was carried out using the Logrank test. A value of *p* < 0.05 was considered statistically significant.

## 3. Results

### 3.1. LPS-Induced Lung Injury in Wild Mice

**Figure 1:**
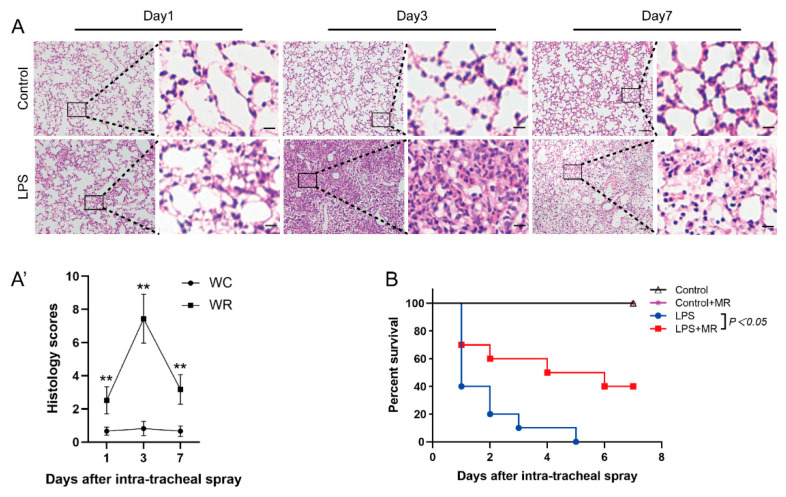
Histopathological changes of lung injury and survival rate of mice over time after LPS administration. (**A**,**A′**) Lung tissues were stained with hematoxylin and eosin and correlated histology scores were counted at days 1, 3, and 7 after LPS (15 mg/kg) or saline administration. Values are expressed as the means ± SE, *n* = 5 in each group. Scale bars: 100 μm (original micrograph), 15 μm (enlarged). (**B**) Survival curve after LPS administration (25 mg/kg). *n* = 10 in each group. ** *p* < 0.01 compared with control group.

The lungs were harvested at days 1, 3, and 7 after LPS administration. Pathological detection showed that inflammatory cell infiltration was found at all observed time points (*p* < 0.01). However, the inflammatory cell infiltration peaked at day 3 and then faded away over time (Figure 1A(A′)). In addition, edema, hemorrhage, and atelectasis also peaked at day 3, while these phenomena were rarely found at day 1 and day 7. All these data indicate that LPS-induced lung injuries were initiated at day 1, peaked at day 3, and then attenuated over time. Therefore, day 3 after LPS administration was chosen as the observed time point in the following studies.

### 3.2. MR Attenuated LPS-Induced Lung Injury

MR improved the survival rate of ALI mice which were administrated with 25 mg/kg LPS. As shown in Figure 1B, 60% of the mice died at day 1 and none survived to day 7 after LPS administration in the LPS group. In the MR group, 30% of the mice died at day 1 and 40% of the mice were still alive at day 7 after LPS administration (*p* < 0.05). It indicates that MR improved the prognosis of LPS-induced lung injury mice.

**Figure 2:**
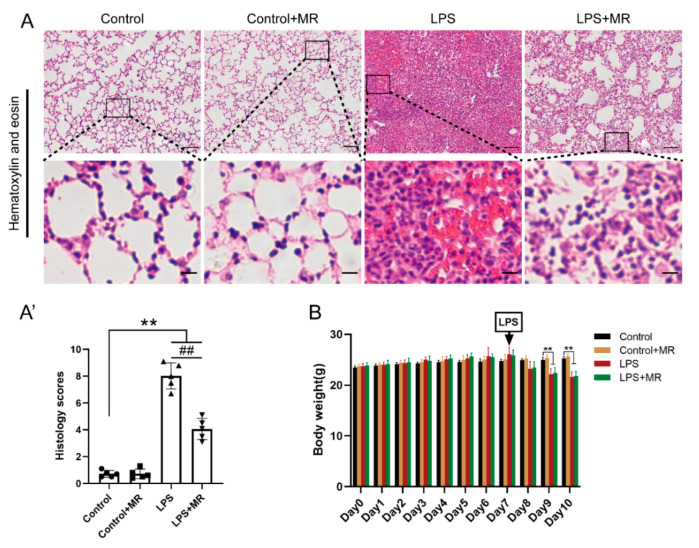
MR attenuated LPS-induced lung injury without malnutrition in mice. (**A**,**A′**) Lung tissues were stained with hematoxylin and eosin and correlated histology scores were counted at day 3 after LPS administration. (**B**) Dynamic alterations of body weight from day 7 before LPS administration to day 3 after LPS administration. Values are expressed as the means ± SE; *n* = 5 in each group (**A′**); *n* = 6 in each group (**B**). ** *p* < 0.01 compared with control group. ## *p* < 0.01 compared with LPS group. Scale bars: 100 μm (original micrograph), 15 μm (enlarged).

In addition, MR attenuated LPS-induced inflammatory cell infiltration, edema, hemorrhage, and atelectasis compared with that of the LPS group (Figure 2A(A′)) (*p* < 0.01). Moreover, the body weights of the mice in each group had no significant difference before the LPS challenge. After LPS administration, the body weights in the LPS and LPS + MR groups decreased dramatically compared with the control group (*p* < 0.01). However, there was no significant difference between the LPS and LPS + MR groups (*p* > 0.05) (Figure 2B). These data indicated that MR attenuated LPS-induced lung injury and did not lead to malnutrition in wild mice.

### 3.3. MR Ameliorated LPS-Induced Alveolar Epithelial Cell Injuries

**Figure 3:**
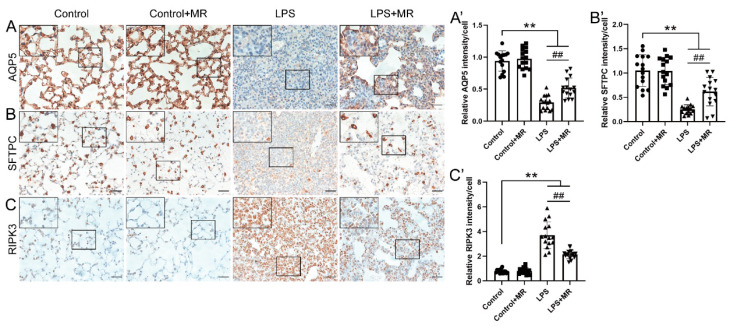
MR ameliorated LPS-induced alveolar epithelial cells injuries. Immunohistochemical staining and quantification of relative intensity of AQP5 (**A**,**A′**), SFTPC (**B**,**B′**), RIPK3 (**C**,**C′**) at day 3 after LPS administration. Values are expressed as the means ± SE; *n* = 15 alveoli from 5 mice (**A′**–**C′**). ** *p* < 0.01 compared with control group. ## *p* < 0.01 compared with the LPS group. Scale bars: 20 μm (original micrograph), 5 μm (enlarged).

Alveolar epithelial is composed of two epithelial cell types: squamous alveolar type 1 cells (AT1) that mediate gas exchange and cuboidal alveolar type 2 cells (AT2) that secrete surfactant to prevent alveolar collapse. AQP5 is an apical protein and displays a polarized and cell-specific distribution in the AT1 cell and thus acts as an AT1 cell marker, while SFTPC is regarded as the specific marker of the AT2 cell phenotype [30,31,32]. Immunochemistry staining showed that LPS instillation led to a decrease in AQP5 and SFTPC expression (*p* < 0.01), which indicated that the alveolar epithelial cells were damaged after a LPS challenge (Figure 3A,B). Moreover, the AQP5 and SFTPC expression increased in the MR group compared with the LPS group (*p* < 0.01). Furthermore, the receptor-interacting protein kinase 3 (RIPK3), a critical regulator of necroptosis and a biomarker of inflammation-induced cell death [33,34] was found to be increased after LPS administration, and MR partially reversed this change (*p* < 0.01) (Figure 3C). These data indicated that MR attenuated LPS-induced injuries of alveolar epithelial cells.

### 3.4. MR Attenuated LPS-Induced Inflammatory Response through TLR4/NF-κB/NLRP3 Signaling

**Figure 4:**
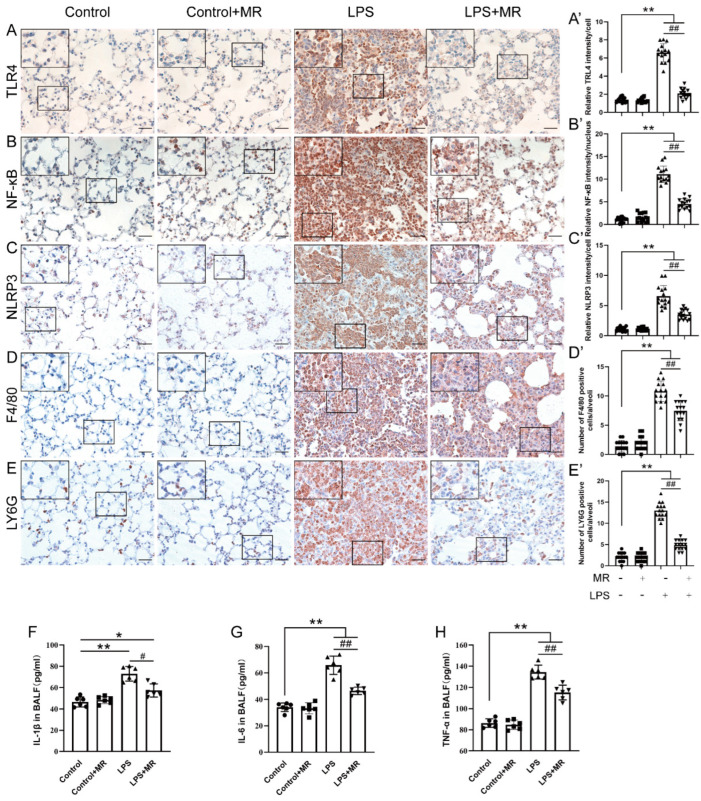
MR attenuated LPS-induced inflammatory response through TLR4/NF-κB/NLRP3 signaling. Immunohistochemical staining and quantification of relative intensity of TLR4 (**A**,**A′**), NF-κB (**B**,**B′**), NLRP3 (**C**,**C′**), F4/80 (**D**,**D′**), LY6G (**E**,**E′**) at day 3 after LPS administration. IL-1β (**F**), IL-6 (**G**) and TNF-α (**H**) levels of bronchoalveolar lavage fluid were determined by ELISA. Values are expressed as the means ± SE; *n* = 15 alveoli from 5 mice (**A′**–**E′**); *n* = 6 in each group (**F**–**H**). * *p* < 0.05, ** *p* < 0.01 compared with control group. # *p* < 0.05, ## *p* < 0.01 compared with LPS group. Scale bars: 20 μm (original micrograph), 5 μm (enlarged).

TLR4/NF-κB/NLRP3 is a common signaling pathway in the LPS-induced inflammatory response [35,36]. Immunochemistry analysis showed that LPS administration increased TLR4 and NLRP3 expression, and nuclear translocation of NF-κB in our ALI model (*p* < 0.01) (Figure 4A–C). Meanwhile, the expression of macrophages marker F4/80 and neutrophils marker LY6G also markedly increased (*p* < 0.01) (Figure 4D,E). These data suggested that the LPS challenge triggered an inflammatory response in the lungs. In line with this, the IL-1β, IL-6 and TNF-α levels in BALF also increased (Figure 4F–H). However, MR partially reversed these changes (*p* < 0.05) (Figure 4A–H), which suggested that LPS-induced TLR4/NF-κB/NLRP3 pathway activation and subsequent inflammatory response were attenuated by MR.

### 3.5. MR Increased H_2_S Production by Upregulating CSE but Not CBS/MST in LPS-Induced ALI Mice

**Figure 5:**
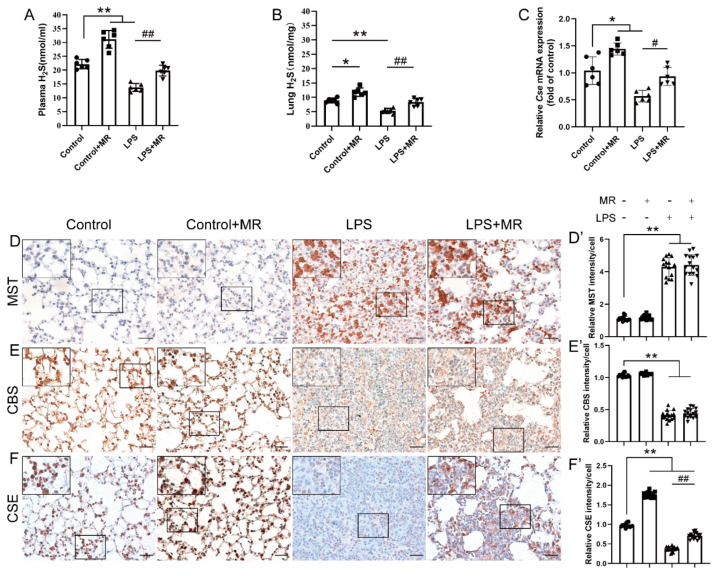
MR increased H_2_S production by upregulating CSE but not CBS/MST in LPS-induced ALI mice. (**A**,**B**) The levels of H_2_S in the plasma and lungs were determined respectively according to the protocol. (**C**) The mRNA levels of CSE in the lungs were analyzed by qPCR. Immunohistochemical staining and quantification of relative intensity of MST (**D**,**D′**), CBS (**E**,**E′**), CSE (**F**,**F′**) at day 3 after LPS administration. Values are expressed as the means ± SE; *n* = 6 in each group (**A**–**C**); *n* = 15 alveoli from 5 mice (D′–F′). * *p* < 0.05, ** *p* < 0.01 compared with control group. # *p* < 0.05, ## *p* < 0.01 compared with LPS group. Scale bars: 20 μm (original micrograph), 5 μm (enlarged).

Based on the previous studies showing that H_2_S plays a vital role in various disease models upon MR intervention, we further explored the effects of MR on H_2_S production and the corresponding enzymes that contribute to H_2_S production in this model. We found that H_2_S levels decreased after LPS instillation at day 3 both in the plasma and lung, and MR intervention reversed these changes (*p* < 0.01) (Figure 5A,B).

To date, there are three enzymes—CSE, CBS and MST—that contribute to H_2_S production in mammals. Thus, the expression of these enzymes were detected by immunochemistry in this study. As shown in Figure 5D–F, the LPS challenge increased MST expression, while it lowered the CBS and CSE expression remarkably (*p* < 0.01). MR intervention had almost no effects on CBS and MST expression compared with that of the LPS group (*p* > 0.05). However, the CSE expression was strikingly increased upon MR intervention (*p* < 0.01), which was consistent with the result of qPCR quantification analysis that MR-reversed the decrease in mRNA expression of CSE after the LPS challenge (Figure 5C). These data indicated that MR improved H_2_S production via upregulating CSE expression in this ALI model.

### 3.6. Inhibition of CSE Eliminated the Protective Effects of MR on LPS-Induced ALI and Reversed H_2_S Levels Increase upon MR

**Figure 6:**
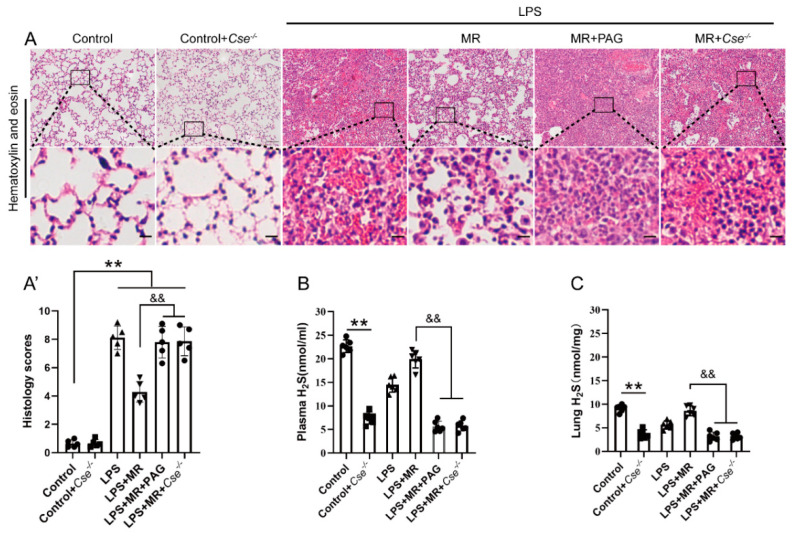
Inhibition of CSE eliminated the protective effects of MR on LPS-induced ALI and reversed H_2_S levels increase upon MR. (**A**,**A′**) Lung tissues were stained with hematoxylin and eosin and correlated histology scores were counted at day 3 after LPS administration. (**B**,**C**) The levels of H_2_S in the plasma and lungs were determined respectively according to the protocol. Values are expressed as the means ± SE; *n* = 5 in each group (**A′**); *n* = 6 in each group (**B**,**C**). ** *p* < 0.01 compared with control group. && *p* < 0.01 compared with LPS + MR group. Scale bars: 100 μm (original micrograph), 15 μm (enlarged).

To explore the roles of CSE in the process, a specific CSE inhibitor PAG was used to detect the effects on MR in LPS-induced ALI. As shown in Figure 6A, PAG treatment eliminated the protective roles of MR that attenuated LPS-induced inflammatory cell infiltration, edema, hemorrhage, and atelectasis (*p* < 0.01). Meanwhile, PAG treatment led to a decrease in H_2_S levels in the plasma and lung compared with that of the LPS + MR group (*p* < 0.01) (Figure 6B,C). Moreover, to further verify the changes of PAG derived from inhibiting CSE, *Cse*^−/−^ mice were employed to repeat these experiments, and similar results were observed as shown in Figure 6A–C. These results suggested that MR attenuated LPS-induced ALI via the modulation of CSE expression, and the inhibition of CSE reduced the H_2_S levels.

### 3.7. Exogenous H_2_S Administration Mimicked the Protective Effects of MR in Cse^−^^/^^−^ Mice after LPS Administration

**Figure 7:**
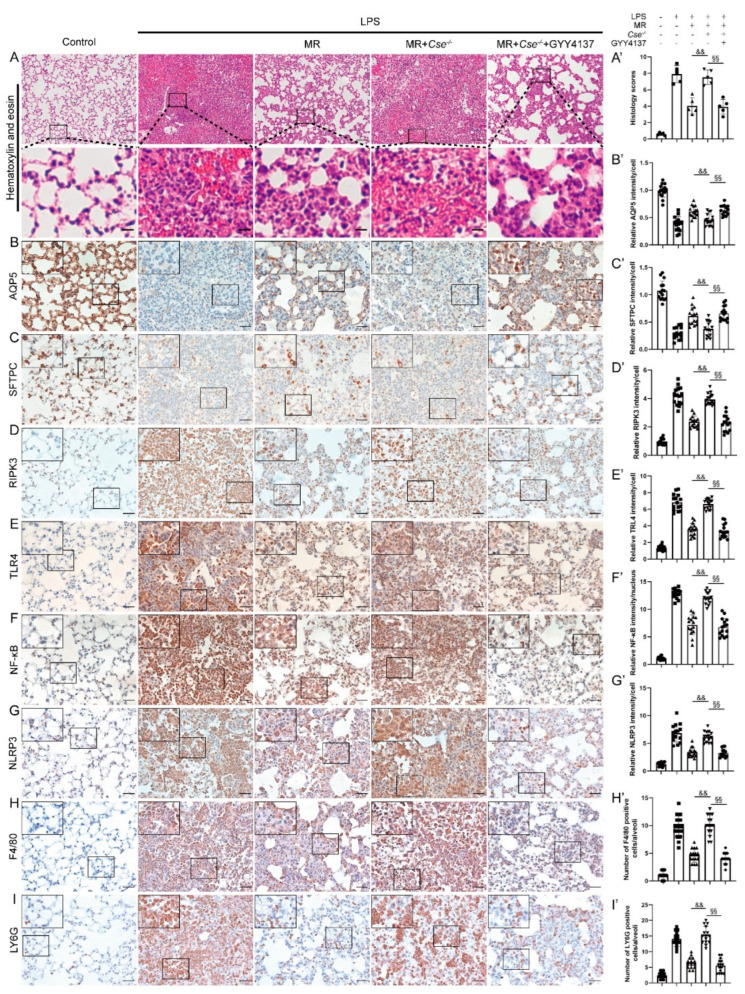
Exogenous H_2_S administration mimicked the protective effects of MR in *Cse*^−/−^ mice after LPS administration. (**A**,**A′**) Lung tissues were stained with hematoxylin and eosin and correlated histology scores were counted at day 3 after LPS administration. Immunohistochemical staining and quantification of relative intensity of AQP5 (**B**,**B′**), SFTPC (**C**,**C′**), RIPK3 (**D**,**D′**), TLR4 (**E**,**E′**), NF-κB (**F**,**F′**), NLRP3 (**G**,**G′**), F4/80 (**H**,**H′**), LY6G (**I**,**I′**) at day 3 after LPS administration. Values are expressed as the means ± SE; *n* = 5 in each group (**A′**); *n* = 15 alveoli from 5 mice (**B′**–**I′**). && *p* < 0.01 compared with LPS + MR group. §§ *p* < 0.01 compared with LPS + MR + *Cse*^−/−^ group. Scale bars: 100 μm (original micrograph), 15 μm (enlarged) (**A**); 20 μm (original micrograph), 5 μm (enlarged) (**B**–**I**).

We further explored the effects of H_2_S on the MR’s protection against LPS-induced lung injuries. GYY4137, a slow-releasing donor of H_2_S was employed to determine if H_2_S was necessary for MR-mediated lung protection against LPS. As shown in Figure 7, MR’s protection against LPS induced lung injuries and inflammatory response via the inhibition of TLR4/NF-κB/NLRP3 pathway were not found in *Cse*^−/−^ mice (*p* < 0.01), whereas GYY4137 treatment restored these protective effects of MR in *Cse*^−/−^ mice (Figure 7A–I) (*p* < 0.01). These data suggested that H_2_S played vital roles in the process that MR protected against LPS-induced ALI, and exogenous H_2_S treatment alone mimicked the protective effects of MR in *Cse*^−/−^ mice after LPS administration.

Taken together, MR attenuated LPS-induced ALI via upregulating the CSE/H_2_S pathway, inhibiting the inflammatory response via the TLR4/NF-κB/NLRP3 pathway, and ameliorating alveolar epithelial cell injuries.

## 4. Discussion

In the current study, we demonstrated that MR mitigates LPS-induced lung injury, which is characterized by a remarkably inflammatory response, inflammatory cell infiltration and alveolar epithelial cell injuries, and improves the prognosis of ALI mice. CSE deficiency eliminates the protective effects of MR and reduces the H_2_S production as well. Exogenous H_2_S treatment alone restores MR’s protection effects in *Cse^−/−^* mice. Thus, in summary, these results suggest that CSE/H_2_S signaling plays an important protective role in LPS-induced ALI.

Methionine is an essential amino acid (EAA). It works as a receptor ligand that is involved in various biochemical and physiological processes [37]. Under normal physiological conditions, methionine is converted to cysteine for further utilization by cells through the transsulfuration pathway (TSP), which is a metabolic pathway that transfers sulfur from homocysteine to cysteine. It is interesting to note that MR reduces the intake of methionine that leads to a decrease in cysteine production and then compensatory de novo synthesis of cysteine, thus increasing the production of two key enzymes of TSP, namely CSE and CBS. It is an evolutionarily conserved response to MR from yeast to mammals [7,38]. In line with this, our data also showed that MR significantly increased the expression of CSE but not CBS in LPS-induced ALI mice, indicating that the main target of MR in TSP was CSE in our ALI model. In addition, several sulfur metabolites, including taurine, GSH and the gaseous signaling molecule H_2_S, are also produced in the transsulfuration pathway. Although all of them have potential protection against LPS-induced ALI, our data showed that the addition of H_2_S alone almost mimicked the protective effects of MR. It suggests that H_2_S is the most important metabolite of TSP for the protective effects of MR, but further studies are needed to investigate the roles of GSH and taurine.

TLR4 is a key pattern-recognition receptor that mediates LPS signaling into cells and triggers an inflammatory response of the innate immunity. It is expressed in many types of cells, such as lymphocytes, macrophages, neutrophils, endothelial, and epithelial cells [39,40]. After recognizing LPS, TLR4 initiates MyD88-dependent NF-κB activation and then provides the signal required for the activation of inflammasomes, ultimately leading to excessive inflammation and even endotoxin shock [41,42]. Inflammasomes consist of a number of nucleotide-binding oligomerization domain (NOD)-like receptor (NLR) family members, which are responsible for the activation of inflammatory cytokines [43,44]. NLRP3 is one of the members that has been studied the most as it responds to a multitude of external stimuli, including sepsis. NLRP3 senses reactive oxygen species (ROS), recruits apoptosis-associated speck-like protein containing a caspase activation and recruitment domain (ASC) and pro-caspase-1, and then forms an active inflammasome complex which subsequently induces the secretion of potent inflammatory cytokines such as IL-1β and even cell death via pyroptosis [45]. Taken together, it is consistent with our data that the TLR4/NF-κB/NLRP3 pathway plays an important role in LPS-induced ALI.

Accumulating evidence suggests that H_2_S can inhibit the activation of the TLR4/NF-κB/NLRP3 pathway [40,46,47]. Previous studies have reported that TLR4 and differentiation factor-2 (MD-2) form a heterodimer in the extracellular domain of TLR4, which is essential for LPS recognition [48]. After that, Toll-like receptors (TLRs) recruit Toll/interleukin-1 receptor (TIR)-domain-containing adaptor proteins, such as MyD88, which interact with TLRs via TIR-TIR interaction in the intracellular domain to form a dimer to deliver the information of pathogen-associated molecular patterns (PAMPs) to downstream signaling and ultimately activate the transcription factor NF-κB [49]. TLR antagonists such as Eritoran and lipid IVa, which are, respectively, analogs of the LPS and LPS precursors, can antagonize TLR4 signaling by binding to the TLR4-MD-2 complex. Accordingly, TLR antagonists are deemed to interfere with the dimerization of TLRs as a general rule [50]. In the current study, H_2_S was found to inhibit the activation of TLR4/NF-κB/NLRP3 pathway and it may be the case that H_2_S attenuated LPS-induced ALI by interfering with the dimerization of TLR4. Besides, it has been reported that H_2_S also inhibits the activity of NF-κB by sulfhydrating the p65 subunit of NF-κB at cysteine-38 [51]. Moreover, H_2_S can also alleviate ALI by other pathways, including preventing neutrophils from infiltrating the lungs, inhibiting autophagy via the PI3K/Akt/mTOR pathway, and suppressing oxidative stress [52,53,54]. A number of studies have demonstrated that exogenous H_2_S is beneficial in various models of lung injury, such as ventilator-induced lung injury (VILI), endotoxin-induced ALI, oleic acid-induced ALI, oxidative lung injury, and trauma-induced ALI [55,56,57,58]. All these points indicate that H_2_S indirectly or directly protects LPS-induced lung injury.

The use of exogenous H_2_S to prevent organ injuries is still in the pre-clinical study stage, and there are many questions (e.g., the route, dosing, and timing of administration) that need to be answered. However, unlike the application of exogenous H_2_S, MR alleviated LPS-induced ALI in mice by upregulating the endogenous H_2_S levels via the modulation of the concentration of substrate in TSP. Furthermore, as an alternative method to enforce the restriction of the calorie intake, MR does not lead to malnutrition, and it is true that the body weight was not reduced throughout our experiments. Therefore, it is likely that the MR strategy can be further developed for clinical use.

## 5. Conclusions

**Figure 8:**
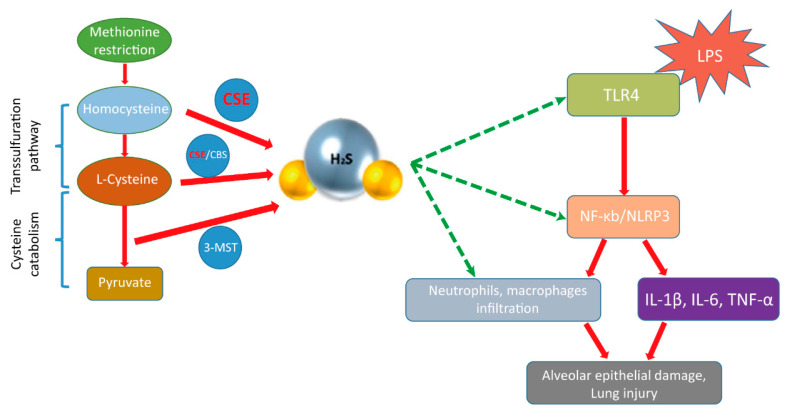
The scheme of the mechanism for the protective effect of MR on LPS-induced ALI.

The current study showed that MR prevents LPS-induced ALI, including the inflammatory response, inflammatory cell infiltration, and alveolar epithelial cell injuries without malnutrition via upregulating the CSE/H_2_S pathway, which is likely associated with inhibition of the TLR4/NF-κB/NLRP3 pathway (Figure 8). This study suggests that developing MR for clinical applications in critically ill patients may be a promising strategy.

## Data Availability

Not applicable.

## Supplementary Materials

The following supporting information can be downloaded at: https://www.mdpi.com/article/10.3390/nu14020322/s1, Table S1: The ingredients of the experimental diets; Table S2: The primary antibodies of immunochemistry.
