# Correction: Duan et al. Methionine Restriction Prevents Lipopolysaccharide-Induced Acute Lung Injury via Modulating CSE/H_2_S Pathway. *Nutrients* 2022, *14*, 322

**DOI:** 10.3390/nu16121915

**Published:** 2024-06-18

**Authors:** Jiaxiang Duan, Lunli Xiang, Zhen Yang, Li Chen, Jianteng Gu, Kaizhi Lu, Daqing Ma, Hailin Zhao, Bin Yi, Hongwen Zhao, Jiaolin Ning

**Affiliations:** 1 Department of Anesthesia, Southwest Hospital, Third Military Medical University, Chongqing 400038, China; duanjiaxiang@tmmu.edu.cn (J.D.); yangzhen@tmmu.edu.cn (Z.Y.); jiantenggu@tmmu.edu.cn (J.G.); lukaizhi@tmmu.edu.cn (K.L.); 2 Department of Nephrology, Southwest Hospital, Third Military Medical University, Chongqing 400038, China; xianglunli@tmmu.edu.cn; 3 Department of Breast and Thyroid Surgery, Southwest Hospital, Third Military Medical University, Chongqing 400038, China; chenlili@tmmu.edu.cn; 4 Division of Anaesthetics, Pain Medicine and Intensive Care, Department of Surgery and Cancer, Faculty of Medicine, Imperial College London, Chelsea and Westminster Hospital, London SW10 9NH, UK; d.ma@imperial.ac.uk (D.M.); hailin.zhao06@imperial.ac.uk (H.Z.)

## Error in Figure

In the original publication [1], there were mistakes in the published version of Figure 7A (LPS group), 7D (control group), 7F (LPS group), 7G (LPS group); the authors uploaded the wrong images during final proofreading. In the correction, they have been replaced. The corrected version of Figure 7 appears below.

**Figure 7:**
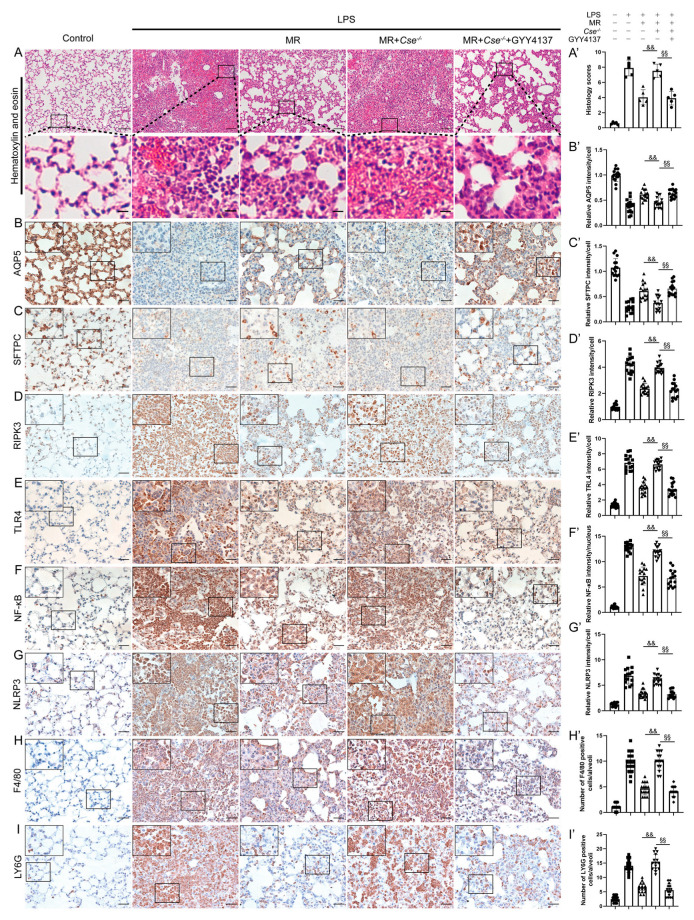
Exogenous H_2_S administration mimicked the protective effects of MR in *Cse^−/−^* mice after LPS administration. (**A**,**A’**) Lung tissues were stained with hematoxylin and eosin and correlated histology scores were counted at day 3 after LPS administration. Immunohistochemical staining and quantification of relative intensity of AQP5 (**B**,**B’**), SFTPC (**C**,**C’**), RIPK3 (**D**,**D’**), TLR4 (**E**,**E’**), NF-κB (**F**,**F’**), NLRP3 (**G**,**G’**), F4/80 (**H**,**H’**), LY6G (**I**,**I’**) at day 3 after LPS administration. Values are expressed as the means ± SE; n = 5 in each group (**A’**); n = 15 alveoli from 5 mice (**B’**–**I’**). && *p* < 0.01 compared with LPS + MR group. §§ *p* < 0.01 compared with LPS + MR + *Cse^−/−^* group. Scale bars: 100 μm (original micrograph), 15 μm (enlarged) (**A**); 20 μm (original micrograph), 5 μm (enlarged) (**B**–**I**).

The authors apologize for any inconvenience caused and state that the scientific conclusions are unaffected. This correction was approved by the Academic Editor. 
