# Supplementary material for: Methionine Restriction Prevents Lipopolysaccharide-Induced Acute Lung Injury via Modulating CSE/H2S Pathway
Source: Nutrients. 2022 Jan 13;14(2):322. doi: 10.3390/nu14020322 (PMC8777780; doi:10.3390/nu14020322)
Supplement: Supplementary file 1 [file nutrients-14-00322-s001.zip › nutrients-1513928-supplementary.pdf]

Supplementary Table S1 The ingredients of the experimental diets

Diet	Control methionine diet (0.86% methionine)	Methionine restriction diet (0.43% methionine)
Amino acid concentrations		
Arginine	0.93%	0.93%
Glycine	2.31%	2.31%
Histidine	0.27%	0.27%
Isoleucine	0.82%	0.82%
Leucine	1.11%	1.11%
Lysine	1.15%	1.15%
Phenylalanine	1.16%	1.16%
Threonine	0.82%	0.82%
Tryptophan	0.18%	0.18%
Valine	0.82%	0.82%
Cysteine	0%	0%
**Methionine**	**0.86%**	**0.43%**
Corn starch	47.97%	48.40%
Sucrose	20%	20%
Corn oil	8%	8%
Maltodextrin	5%	5%
Cellulose	5%	5%
Glutamic acid, vitamin and mineral mix, choline	3.6%	3.6%

Supplementary Table S2 The primary antibodies of immunochemistry

Primary antibody	Catalog no.	Host species	dilution	Brand
Aquaporin-5 (AQP5)	ab78486	rabbit polyclonal	1:500	Abcam, Cambridge, United Kingdom
Cystathionine-beta-synthase (CBS)	ab135626	rabbit polyclonal	1:50	Abcam, Cambridge, United Kingdom
Cystathionine-gamma-lyase (CSE)	ab151769	rabbit polyclonal	1:500	Abcam, Cambridge, United Kingdom
Adhesion G protein-coupled receptor E1 (F4/80)	ab16911	rat monoclonal	1:50	Abcam, Cambridge, United Kingdom
lymphocyte antigen 6 complex (LY6G)	ab238132	rabbit monoclonal	1:50	Abcam, Cambridge, United Kingdom
3-mercaptopyruvate sulfurtransferase (MST)	ab154514	rabbit polyclonal	1:100	Abcam, Cambridge, United Kingdom
NOD-like receptor protein 3 (NLRP3)	bs-10021R	rabbit polyclonal	1:200	Biosynthesis Biotechnology CO., LTD., Beijing, China
Nuclear factor kappa B (NF-κB)	ab16502	rabbit polyclonal	1:1000	Abcam, Cambridge, United Kingdom
Receptor-interacting protein kinase 3 (RIPK3)	bs-3551R	rabbit polyclonal	1:200	Biosynthesis Biotechnology CO., LTD., Beijing, China
Surfactant protein C (SFTPC)	ab211326	rabbit monoclonal	1:2000	Abcam, Cambridge, United Kingdom
Toll-like receptors 4 (TLR4)	ab13556	rabbit polyclonal	1:100	Abcam, Cambridge, United Kingdom
